# Pediatric Long Bone Fractures After Dog Bites: A Case Series and Systematic Review

**DOI:** 10.7759/cureus.47230

**Published:** 2023-10-17

**Authors:** Purav S Brahmbhatt, Isra Taha, Chadi Nahal, Sivashanmugam Raju

**Affiliations:** 1 Department of Orthopedic Surgery, Saint Louis University School of Medicine, St. Louis, USA

**Keywords:** dog bite, animal bite, open fracture, wound closure, pediatric

## Abstract

This study presents a case series and systematic review of pediatric patients who sustained long bone fractures following dog bites.

A systematic review of the studies on “pediatric fracture dog bite” based on a search of PubMed and OVID Medline databases was performed by adhering to PRISMA guidelines. Articles in English describing pediatric long bone fractures due to dog bites were included. Studies not differentiating pediatric from adult patients and not describing long bone fractures due to dog bites were excluded. Study characteristics, fracture epidemiology, management decisions, and follow-up data were extracted. Additionally, a seven-year retrospective chart review of cases treated at our level one pediatric trauma center was performed. Data on fracture characteristics, surgical management, choice of antibiotic therapy, and follow-up were collected.

Five studies that met our criteria were analyzed. Pediatric long bone fractures from dog bites were identified in 0.35% (11/3,156) of patients. Such fractures most commonly involved the upper extremity (9/11, 82%). None of the studies described the choice of antibiotics, surgical decision-making, or wound closure preference for an underlying fracture. Our chart review elicited three cases of long bone fractures due to dog bites.

Pediatric long bone fractures after dog bites are a rare injury pattern in the United States. These injuries should be treated as contaminated open fractures, and urgent immunization, intravenous antibiotic administration, wound care, and fracture stabilization should be provided. We recommend meticulous surgical debridement in the operating room, as wounds often probe deep into the bone. Nevertheless, there is much that remains unclear about these injuries. Hence, further research with greater power is needed to improve treatment decisions.

## Introduction

Pediatric dog bite injuries are a significant cause of morbidity and mortality in the United States [1]. Globally, tens of millions of people are injured by dogs, and in the United States alone, dog bite-related injuries account for over 330,000 emergency room (ER) visits annually [2,3]. A dog bite injury may sometimes present as a complex constellation of injuries, involving neurologic, craniofacial, and orthopedic systems [1,4-7]. While pediatric dog bites are well-researched in the fields of emergency medicine, plastic surgery, and neurosurgery, there is scarce data on the orthopedic management of dog bites associated with fractures of the appendicular skeleton. Indeed, while a significant proportion of dog bites require orthopedic attention, there is limited data in the literature about the management of long bone fractures in this setting [8]. Furthermore, while there is an abundance of guidelines on dog bite management, there is little data regarding the management and care of an underlying long bone fracture. Based on our experience at our level one pediatric trauma center, we present three cases of pediatric long bone fractures caused by dog bites and discuss the management strategy employed. Furthermore, we also engage in a systematic review of the current literature regarding the management of pediatric long bone fractures due to dog bites.

Our study involves a retrospective chart review of three cases and a systematic review of the literature. For our retrospective review and case presentation, we analyzed the data available at our level one pediatric trauma center, spanning the period from March 2015 to September 2022, for patients who sustained a dog bite by using the International Classification of Diseases, Tenth Revision (ICD-10) code W54. We identified patients with a documented long bone fracture directly caused by a dog bite. We extracted information regarding the type of fracture, associated injuries, initial orthopedic management, operative and non-operative management, choice of antibiotic therapy, and follow-up. Institutional review board approval was not required for this review.

For our systematic review, we utilized the PRISMA checklist to design our study. We searched for “pediatric fracture dog bite” on the PubMed and OVID Medline databases. Our inclusion criteria were as follows: journal articles in English that included a description of pediatric long bone fractures due to a dog bite. We defined “pediatric” as any person aged 18 years or less at the time of injury. Our definition of “long bone” included the following bones: humerus, radius, ulna, femur, tibia, and fibula. Additionally, we utilized the terms “forearm fracture” and “arm fracture” as analogous to radius, ulna, and humerus fractures, and “leg fracture” or “ankle fracture” as analogous to femur, tibia, and fibula fractures.

Our exclusion criteria were as follows: studies that did not differentiate between adult and pediatric patients, those that did not specifically describe long bone fractures, and studies involving fractures not due to dog bites. We additionally excluded one study because it failed to characterize fractures of the upper extremity and lower extremity as involving long bones versus other bones, such as the bones of the hands or feet. We excluded the term “wrist fracture” in our search as it would lead to difficulty in excluding isolated fractures of the small carpal bones, such as a scaphoid fracture. We analyzed data and extracted information regarding the study design, time frame of the study, number of patients included, number of long bone fractures, treatment regimens, any orthopedic intervention, and the duration of recorded follow-up. All the records were initially screened based on abstracts and titles, followed by a full-text review by an orthopedic surgery resident. Any disputes regarding questionable exclusion were resolved by the attending orthopedic surgeon.

## Case presentation

Case 1

**Figure 1:**
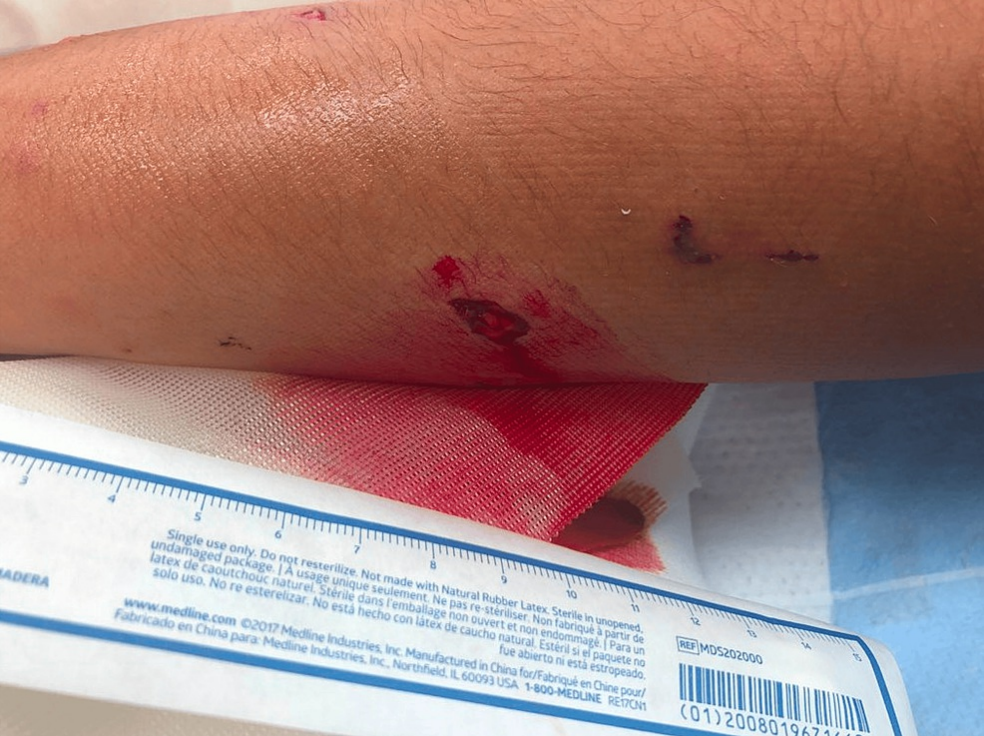
Clinical image of puncture wounds of the dorsal distal forearm due to a dog bite

A 12-year, seven-month-old male with no relevant past medical history presented to the emergency department at an outside hospital after an attack by a known pit bull. He had been bitten on the left forearm by the dog. He had sustained a large 1 x 0.5-cm distal volar ulnar laceration with exposed subcutaneous tissue that probed to the bone and two separate puncture wounds on the dorsal distal forearm (Figure 1).

**Figure 2:**
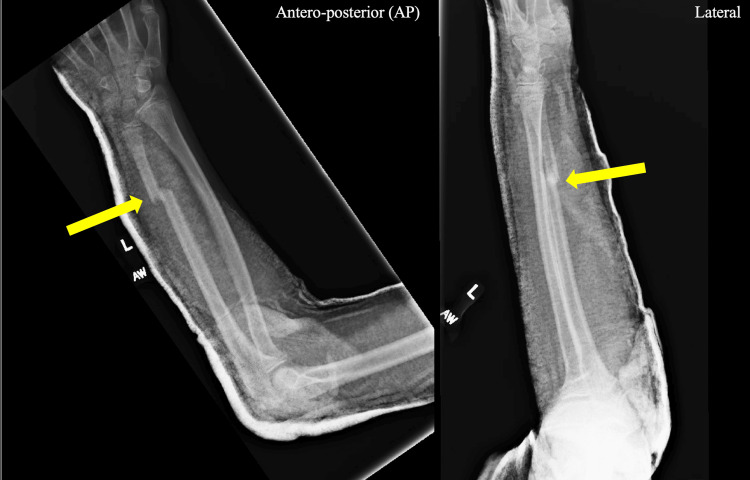
Anteroposterior (AP) and lateral X-ray imaging The arrows point to a displaced distal ulna fracture with an overlying splint

The patient had no neurologic or vascular deficits. Radiographs demonstrated an isolated displaced left ulnar shaft fracture (Figure 2). He had no other associated injuries. The attacking dog was up to date on immunizations and known to the patient.

At the outside hospital, the patient received intravenous ampicillin-sulbactam and local wound care. His arm was splinted, and he was transferred to our level one pediatric emergency department for further evaluation. He was then admitted and administered ampicillin-sulbactam every six hours until formal irrigation and debridement in the operating room the next day. In the operating room, his wound was debrided sharply and explored, and a clear violation of the periosteum with direct communication to the fracture was noted. The wound was closed primarily after proper irrigation and debridement. He was placed in a long arm splint and advised to follow up in one week for a wound check. The patient was discharged with oral amoxicillin clavulanate for 10 days. At the follow-up, the wound did not show any signs of infection, but the ulnar shaft fracture required internal fixation to improve reduction. The patient then underwent uncomplicated flexible elastic intramedullary nailing of his ulnar shaft fracture two weeks after his injury with an additional course of postoperative amoxicillin clavulanate. His fracture healed without any complication, and he underwent hardware removal at roughly eight months postoperatively.

Case 2

**Figure 3:**
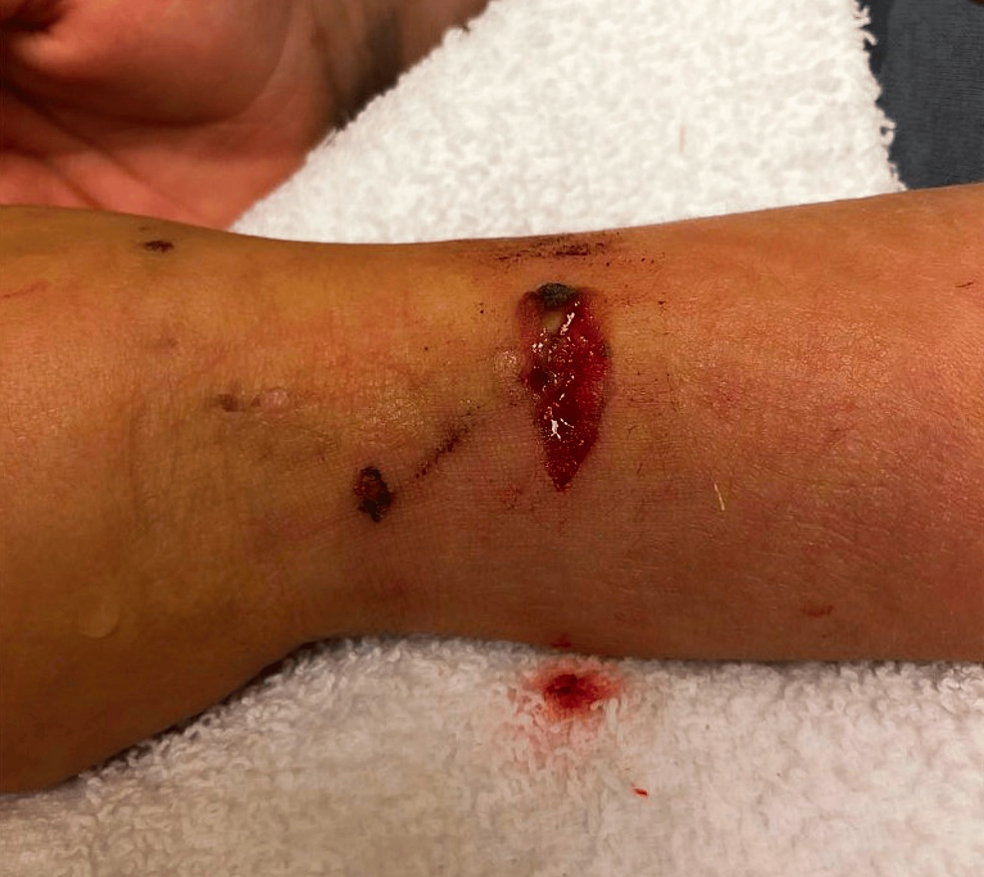
Clinical image of a dorsal distal forearm laceration with exposed subcutaneous tissue

A six-year, seven-month-old male with no relevant past medical history presented to the emergency department at an outside hospital after an attack by a known English bulldog while at a friend’s house. He had sustained a 2 x 1-cm dorsal left distal forearm wound with exposed subcutaneous tissue (Figure 3).

**Figure 4:**
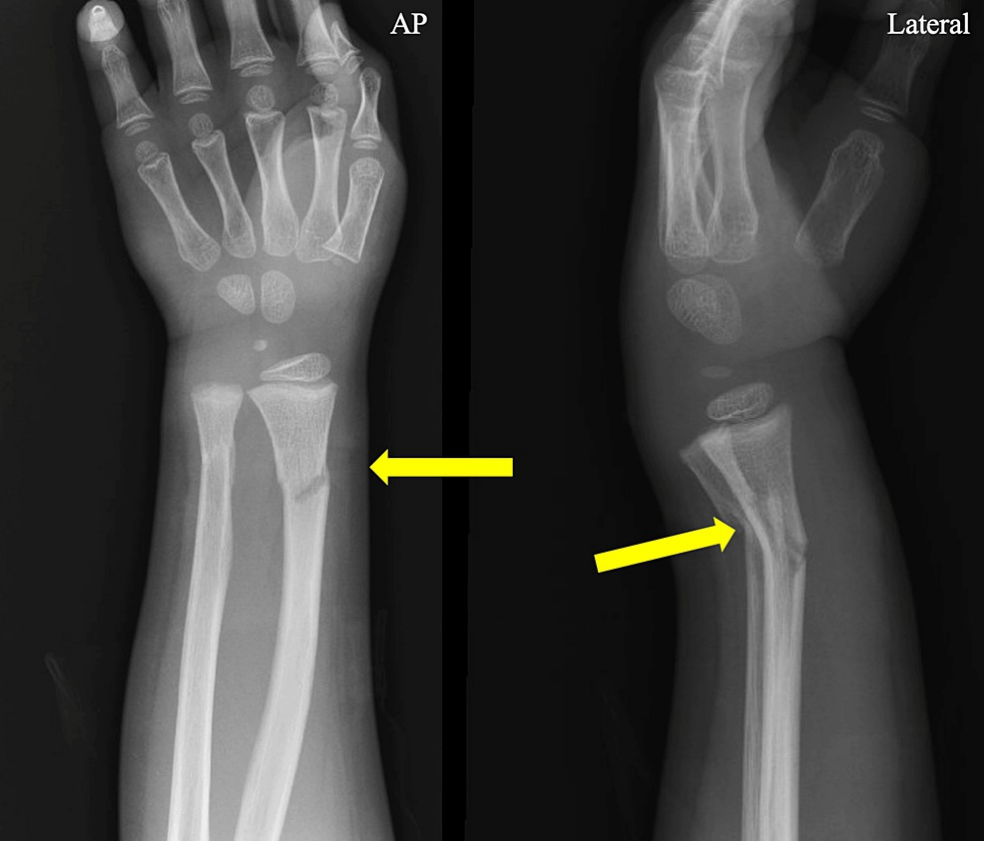
AP and lateral X-ray imaging of the left wrist The arrows point to dorsally angulated, extra-articular distal radius, and ulna fractures

He had also sustained three superficial dog bites to the right leg without fracture. He was found to have a dorsally angulated fracture of the left distal radius and ulna (Figure 4).

The patient had no neurovascular deficits or associated injuries. He and the dog were up to date with immunizations. He was given ampicillin-sulbactam at the outside hospital, his wounds were irrigated and dressed, and his left arm was splinted prior to transfer to our level one pediatric emergency department.

At our facility, his wounds were inspected, irrigated, and dressed under sedation in the emergency department. One wound probed deep to the bone without apparent tendon injury. The wounds were not closed primarily. The distal radius and ulna fractures were closed-reduced and splinted, and the patient was discharged home on oral amoxicillin clavulanate. He was followed up in nine days, and his wound showed no signs of infection. His fracture was then managed with a long arm cast. After appropriate fracture healing was noted by roughly seven weeks, he was discharged with instructions to follow up as needed.

Case 3

**Figure 5:**
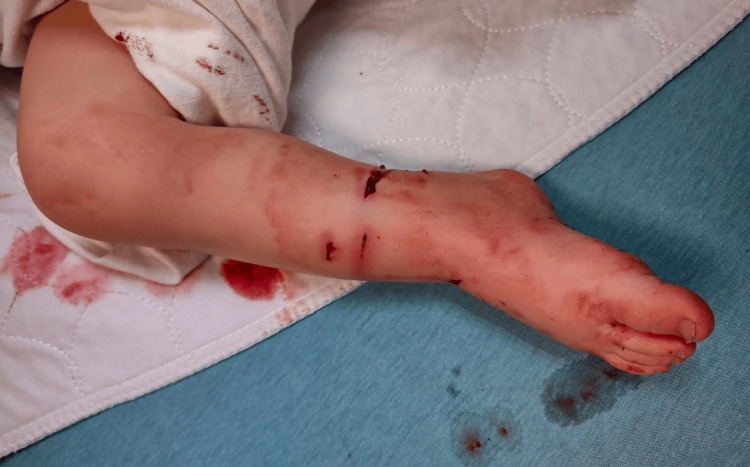
Clinical image of the anteromedial right leg and foot with dog bite wounds

**Figure 6:**
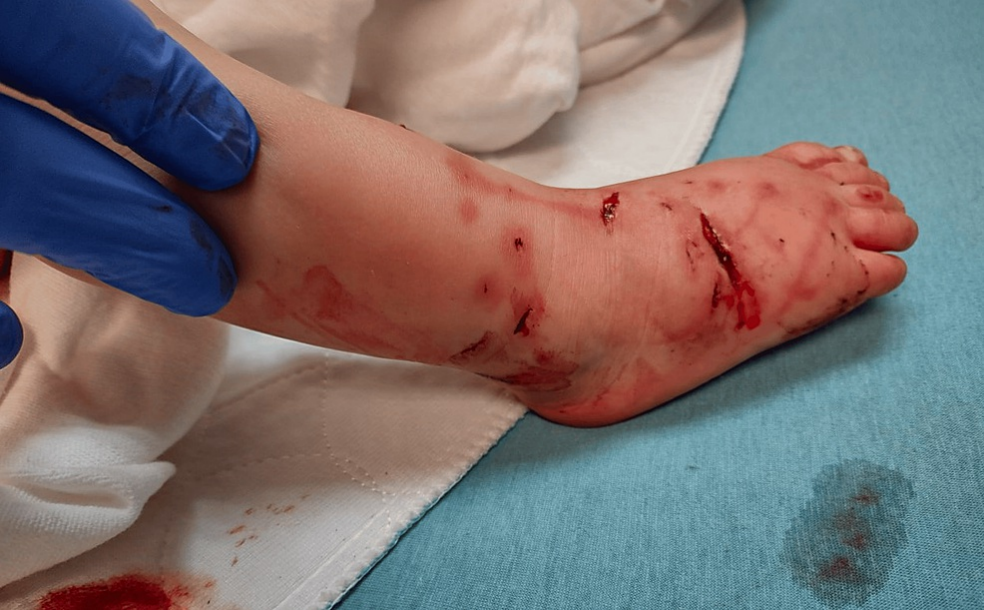
Clinical image of the anterolateral right leg and foot with dog bite wounds

**Figure 7:**
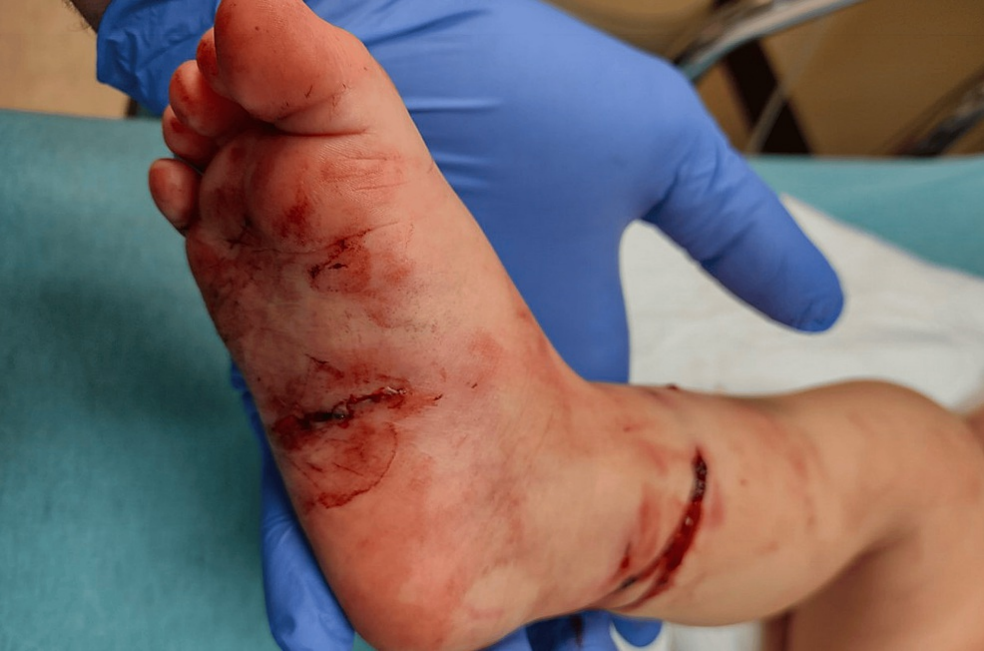
Clinical image of the plantar foot and posteromedial leg with dog bite wounds

An 18-month-old male with a past medical history of Pierre Robin sequence presented from an outside hospital with an open right distal tibia and fibula fracture secondary to a dog bite. He had sustained numerous wounds, including a 1-cm transverse wound at the distal leg tracking to the bone, three punctate wounds at the distal anterolateral leg, a 3-cm transverse dorsal foot wound with exposed tendons, a 1-cm dorsal foot wound deep to subcutaneous tissue, and a 3-cm plantar foot wound deep to subcutaneous tissue (Figures 5, 6, 7).

**Figure 8:**
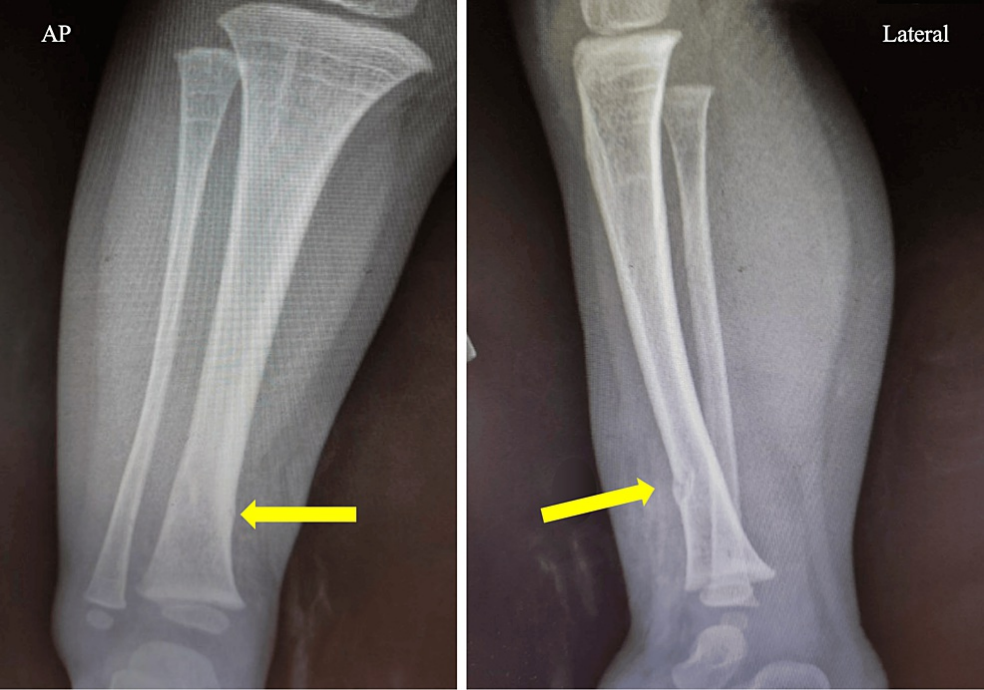
AP view X-ray imaging of the right leg The arrows point to the distal tibial shaft and distal fibula fractures

No neurologic or vascular deficits were noted in the patient, and there were no other associated injuries either. The patient had been given amoxicillin-clavulanate at the outside hospital. At our hospital, plain film imaging on a skeletal survey revealed right distal tibia and fibula fractures without displacement (Figure 8). Initial treatment included irrigation and debridement in the ER with splinting. The patient was administered ampicillin-sulbactam, tetanus vaccine, and immunoglobulin, and started on rabies prophylaxis, as the attacking dog was unknown to the family.

**Figure 9:**
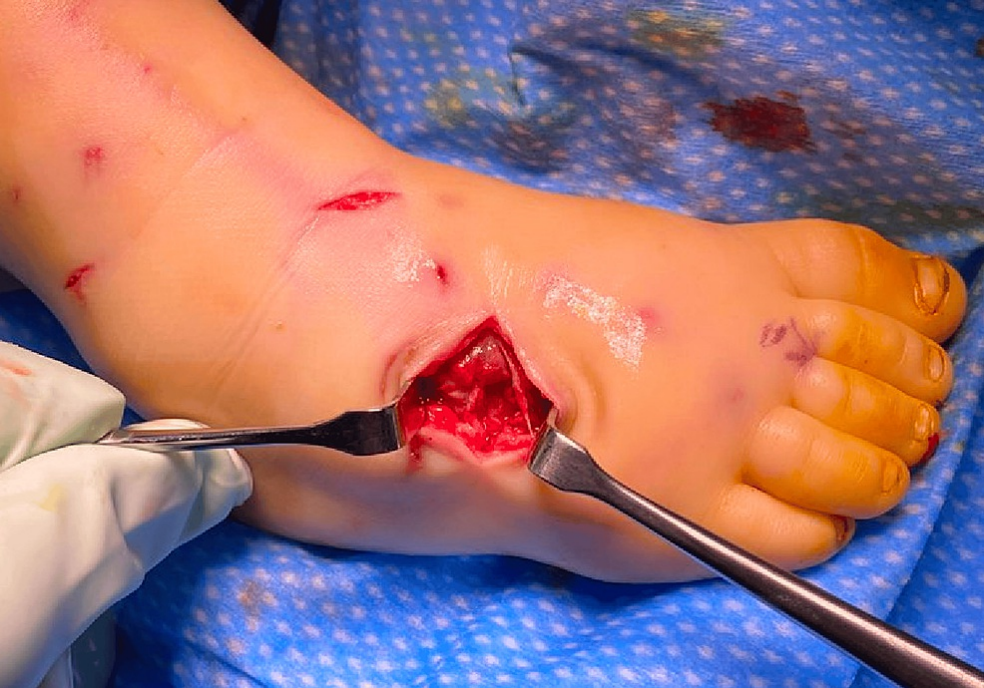
Clinical image showing intraoperative exploration of the dorsal foot wound demonstrating violation of the extensor tendons

The patient was admitted and administered intravenous antibiotics with ampicillin sulbactam every six hours, and scheduled for irrigation and debridement in the OR the following day. Intraoperatively, the patient’s wounds were debrided and sharply excised. The dorsal foot wound was explored, and extensor tendon lacerations were identified and primarily repaired (Figure 9).

**Figure 10:**
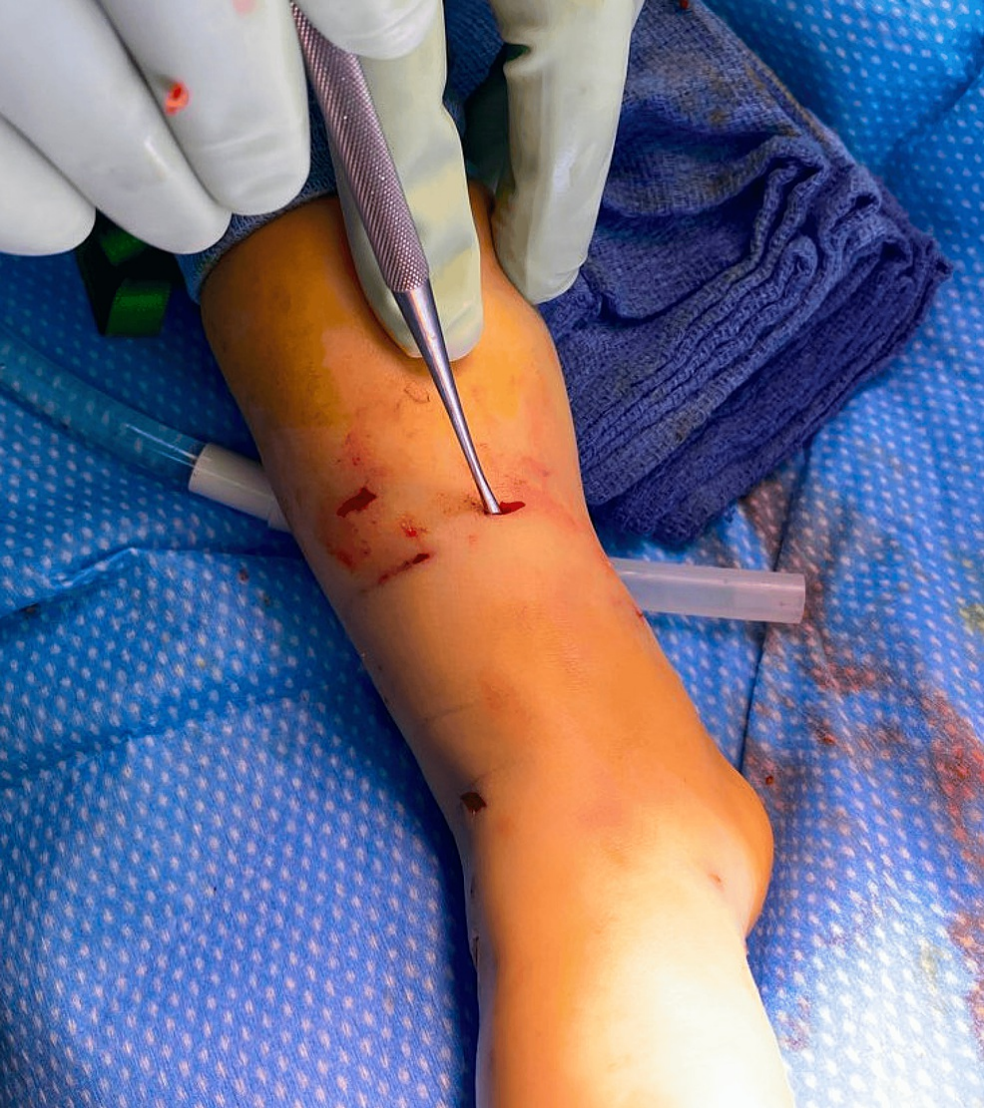
Clinical image of intraoperative probing of the right leg wound with a Freer elevator

**Figure 11:**
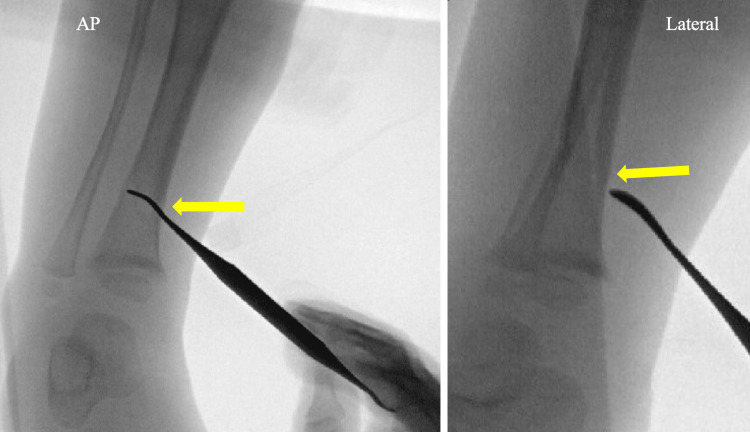
Corresponding intraoperative fluoroscopic imaging The arrows show fracture continuity with the wound via probing

The 1-cm transverse distal leg wound was noted to communicate with the fracture and medullary canal, as shown in Figure 10 and Figure 11. Thereafter, his wounds were dressed, and his fracture was managed with a long leg splint.

The patient was discharged on hospital day three with a prescription for amoxicillin clavulanate twice daily for 10 days. He was transitioned to a long leg cast 10 days following the injury. He received additional doses of rabies post-exposure prophylaxis (PEP) in the coming weeks. Follow-up at seven weeks showed healing on plain film imaging, and the cast was removed. The patient was then discharged from the clinic with instructions to follow up as needed.

## Discussion

**Figure 12:**
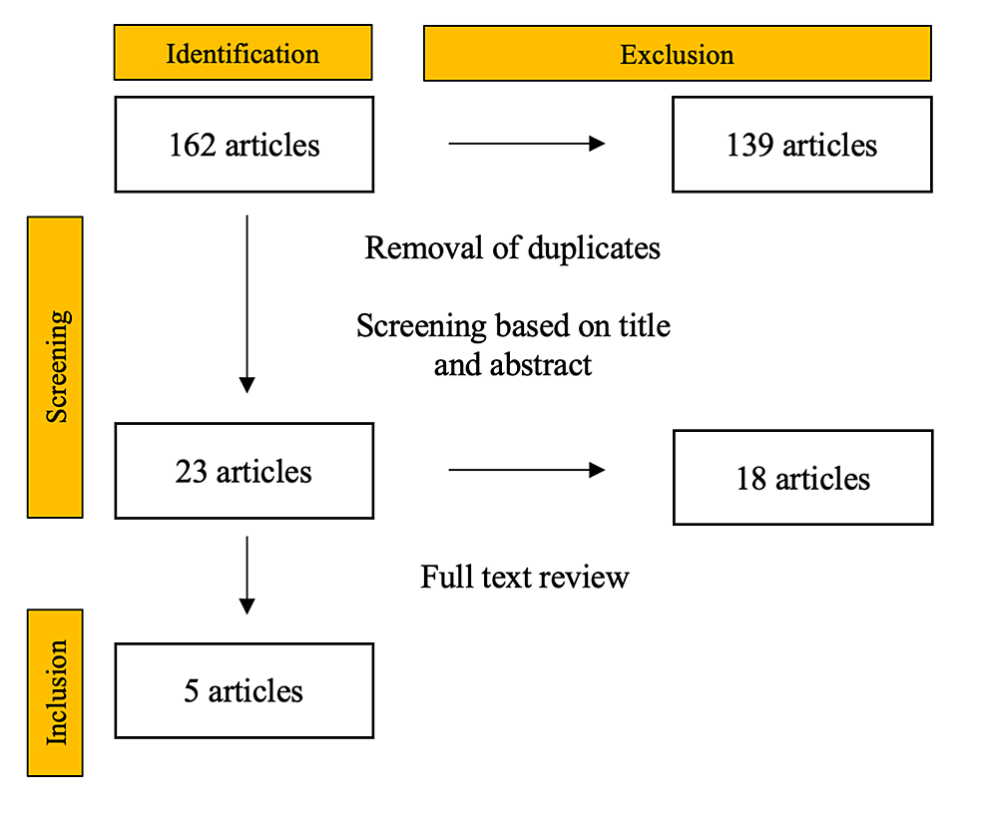
PRISMA flow diagram depicting the selection of studies PRISMA: Preferred Reporting Items for Systematic Reviews and Meta-Analysis

In order to further supplement our case series, we performed a systematic review of the existing literature to assess the current state of awareness about long bone fractures caused by dog bites. In our systematic review, we initially identified a total of 22 articles on PubMed and 142 articles on OVID Medline. After the removal of duplicate studies and screening based on title and abstract, we narrowed it down to six articles from PubMed and 17 from OVID. Ultimately, after full-text reviews, two studies from PubMed and three from OVID were included in the final analysis (Figure 12).

The five studies included were retrospective chart reviews [9-13]. The reviews were conducted at four level one pediatric trauma centers and two pediatric tertiary emergency departments. The average study duration was around 5.45 years (SD: 3 years) with a range of 2-10 years (Table 1).

**Table 1:** Study characteristics and fracture epidemiology

	Authors	Study design	Location	Start date	End date	Time frame (years)	Number of dog bite patients	Number of long bone fractures	Percentage of long bone fractures
1	Borg et al. [9]	Retrospective review	Level 1 pediatric trauma center	2013	2015	2	615	2	0.33%
2	Garvey et al. [10]	Retrospective review	Level 1 pediatric trauma center	10/1/2007	12/31/2013	6.25	282	5	1.77%
3	Golinko et al. [11]	Retrospective review	Level 1 pediatric trauma center	N/A	N/A	4	1616	2	0.12%
4	Lang and Klassen [12]	Retrospective review	Two tertiary pediatric emergency departments	1998	2002	5	287	1	0.35%
5	Reuter Muñoz et al. [13]	Retrospective review	Level 1 pediatric trauma center	7/2007	8/2017	10	356	1	0.28%

The studies involved a total of 3,156 patients who sustained various types of dog bite injuries. Of these, 11 patients had a documented long bone fracture directly related to the dog bite injury, indicating that 0.35% of pediatric dog bites are associated with a long bone fracture. Of these, the most common long bone fractures involved the upper extremity - forearm/radius/ulna: 5/11 (45%) and humerus/arm: 4/11 (36%). The femur and tibia each accounted for 1/11 (9%) fractures (Table 2).

**Table 2:** Management of long bone fractures ORIF: open reduction and internal fixation

	Study	Long bones affected	Treatment	Follow-up	Dog breed	Age/sex of the patient	Antibiotics used
1	Borg et al. [9]	A: femur, B: humerus	Reduction in the operating room	N/A	Pitbull	A: 14-month-old female, B: 24-month-old female	N/A
2	Garvey et al. [10]	A, B, C, D: forearm, E: humerus	N/A	N/A	N/A	N/A	N/A
3	Golinko et al. [11]	A: humerus, B: radius	A: ORIF humerus, B: ORIF radius	N/A	N/A	N/A	N/A
4	Lang and Klassen [12]	A: arm	N/A	N/A	N/A	N/A	N/A
5	Reuter Muñoz et al. [13]	A: tibia	Surgical intervention	N/A	N/A	N/A	N/A

Of note, 3/11 (27%) fractures underwent surgical intervention, and 2/11 (18%) underwent a reduction in the operating room. Data regarding irrigation and debridement in the operating room, staged surgery, and immediate or delayed fixation of fractures was minimal.

Dog bites are a major cause of injury in the United States, and while dog bites are well known to affect the head and neck [14], our study revealed that dog bite fractures affecting the long bone occur in roughly 0.35% of reported pediatric dog injuries. In our review, most of the fractures were observed in the upper extremity, presumably as patients guarded their faces against injury. From our chart review, we found that dog bite fractures present with significant soft tissue injuries that should be addressed.

In our management of these injuries, we paid particular attention to the soft tissue injury along with the underlying fracture. We managed these injuries as contaminated open fractures and administered rabies prophylaxis, tetanus immunization, and a course of intravenous antibiotics as soon as possible [15-17]. We recommend following established public health guidelines regarding criteria for rabies virus vaccination and immunoglobulin injection adjacent to the wound, particularly in cases where the attacking dog is not known to the patient or if the dog has been exhibiting erratic behavior [15,16,18].

Antibiotics should cover the normal oral flora of dogs and provide coverage of human skin flora as well [15,19]. Pending the results on the patient’s allergies, we typically prescribe ampicillin-sulbactam or amoxicillin-clavulanate due to its coverage of Pasteurella multicoda, an organism often associated with dog bite infections [17,19,20]. The literature suggests that the antibiotic treatment for superficial dog bites without obvious cellulitis may involve three to five days of oral antibiotics. However, given the presence of a fracture, and concern for deep structure violation, we prescribe an initial course of intravenous antibiotics until adequate wound care is achieved, followed by discharge with a 7-10-day course of oral antibiotics depending on the severity of injury [17,19]. If the patient has a severe allergy to the aforementioned medications, options including doxycycline and erythromycin [15,17] can be considered. We regularly consult with infectious disease specialists for additional guidance and antibiotic management.

Additionally, we recommend thorough irrigation, debridement, and closed fracture management in the emergency department for these fractures, followed by formal wound irrigation and debridement, with fracture stabilization by implants or casting in the operating room. This management is particularly relevant in pediatric fractures since most fractures are treated conservatively and patients are often sent home with instructions to follow up as outpatients. For irrigation, we recommend the addition of soap and water, povidone iodine, or other antiseptic solutions, as it may help dissipate infectious load and possibly reduce rabies risk [16,21,22].

The decision to close wounds primarily after wound care either in the emergency department or operating room remains a matter of controversy in the literature [23-25]. Overall, there is significant variability in decision-making, which could be related to underlying wound location, depth, and size [23]. The literature suggests that dog bites may be closed primarily after thorough debridement with a similar infection risk as secondary closure; however, studies do note that primary closure is associated with improved scar appearance and cosmesis [25,26]. Scarring is an important psychosocial consideration for the pediatric patient, as scars from traumatic injuries may impact a child’s body image and self-esteem [27,28]. Furthermore, in the presence of fracture, wound management with prolonged immobilization in a splint or a cast is more predictable with primary closure. We typically have these patients return for early follow-ups, for the purpose of splint changes, and frequent wound checks, to monitor for late infection. It should be noted that much of the above literature is not specific to dog bites with underlying fractures and there is scant published data specifically regarding wound closure involving dog bite fractures.

Operative intervention is ultimately a complex decision based on the extent of soft tissue derangement, suspicion about the violation of deeper structures, and underlying long bone fracture patterns. In cases involving fractures, there is a strong suspicion that the attacking dog’s teeth may have violated surrounding tendons, underlying periosteum, and bone. Even small puncture wounds should be probed to identify deeper violations, especially in children (e.g., case 3). In case two, the dorsal forearm wound did probe deep down to the bone. Retrospectively, we would have preferred to debride this patient in the operating room, but the patient had been sent home following the splint application.

Data regarding long-term sequelae of dog bite fractures in long bones is currently scarce. A case report by Ramachandran et al. discussed a pediatric patient with a pathologic fracture of the tibia, due to underlying osteomyelitis, with a remote history of a dog bite in the same region [29]. This emphasizes the fact that any dog bite-related long bone fracture can lead to a deeper infection with associated morbidity. Consideration must also be given as to whether these patients suffered other trauma during the dog attack, in addition to the actual dog bite. A study by Juang et al. reported that “non-dog-bite” related injuries, such as sequelae from being pushed, dragged, or falling after a dog attack, should not be overlooked [30]. We recommend performing a thorough head-to-toe musculoskeletal examination of every patient who has suffered a dog bite to avoid missing occult injuries.

## Conclusions

Dog bites are a major concern in the pediatric population, and underlying fracture management is an area that requires further inquiry. Any wound from a dog bite, regardless of size, should be taken seriously. A wound with an underlying fracture should be treated as a high-grade open fracture with contamination. We recommend irrigation and debridement in the operating room rather than in the emergency department. Additionally, we recommend timely immunizations, intravenous antibiotics, local wound care, and fracture stabilization in the form of internal fixation or casting as needed. Primary wound closure for fractures that have been operatively debrided is advised whenever possible, as it reduces scarring and allows for more predictable wound management in splints and casts. Finally, as our systematic review suggests, the proportion of dog bites that lead to long bone fractures is indeed quite low, and data on this specific topic is minimal in the published literature. Given that our case series is very limited in terms of sample size and follow-up, dedicated multicenter studies with long-term follow-up are required to better identify, and possibly challenge, existing treatment paradigms related to this complex injury. Future collaborative research regarding antibiotic management and operative intervention may help guide improved care for these patients.
